# Direct observation of a non-crystalline state of Li_2_S–P_2_S_5_ solid electrolytes

**DOI:** 10.1038/s41598-017-04030-y

**Published:** 2017-06-23

**Authors:** Hirofumi Tsukasaki, Shigeo Mori, Hideyuki Morimoto, Akitoshi Hayashi, Masahiro Tatsumisago

**Affiliations:** 1 Department of Materials Science, Osaka Prefecture University, Sakai, Osaka, 599-8531 Japan; 2 Graduate School of Science and Technology, Gunma University, Kiryu, Gunma, 376-8515 Japan; 3 Department of Applied Chemistry, Osaka Prefecture University, Sakai, Osaka, 599-8531 Japan

## Abstract

There are two types of solid electrolytes which has been recently expected to be applied to all-solid-state batteries. One is the glasses characterized by an amorphous state. The other is the glass ceramics containing crystalline in an amorphous matrix. However, the non-crystalline state of glasses and glass ceramics is still an open question. It has been anticipated that sea-island and core-shell structures including crystalline nanoparticles have been proposed as candidate models for glass ceramics. Nevertheless, no direct observation has been conducted so far. Here we report the non-crystalline state of Li_2_S–P_2_S_5_ glasses and glass ceramics, and the crystallization behavior of the glasses during heating via direct transmission electron microscopy (TEM) observation. High resolution TEM images clearly revealed the presence of crystalline nanoparticles in an amorphous region. Eventually we suggest that the precipitation and connection of crystalline nanoparticles in an amorphous matrix are key to achieving high ionic conductivity.

## Introduction

Lithium-ion secondary batteries have received a lot of attention in recent years due to their excellent charge-discharge cycle characteristics and high energy density. However, safety concerns exist because they require the use of a flammable organic solvent. To resolve the safety problem, we focused on a sulfide-type all-solid-state battery using a non-flammable inorganic solid electrolyte. Sulfide-based inorganic solid electrolytes are known to be electrochemically stable in a wide potential window and exhibit high lithium ion conductivities due to the high polarizability of sulfide ions^1–6^. Therefore, sulfide solid electrolytes are promising candidates for developing all-solid-state batteries.

Li_2_S–P_2_S_5_ glasses prepared by a mechanical milling technique exhibit high conductivities of over 10^−4^ S cm^−1^ at room temperature^7^. Notably, Li_2_S–P_2_S_5_ glass crystallizes by heat treatment, and its lithium ion conductivities highly depend on the type of precipitated crystalline phases and the composition of Li_2_S^8, 9^. F. Mizuno. *et al*. showed that conductivity was enhanced by crystallization with compositions from 70 to 85 mol% Li_2_S; the obtained glass-ceramics exhibited high conductivity of about 10^−3^ S cm^−1^ at room temperature. With compositions of 70 and 80 mol% Li_2_S, the conductivity enhancement was due to the precipitation of metastable Li_7_P_3_S_11_ and LGPS analog (Li_3.25_P_0.95_S_4_) crystal phases, respectively^10–12^. Therefore, the high conductivities of Li_2_S–P_2_S_5_ glass-ceramics were directly associated with the percent composition of Li_2_S and the type of precipitated crystalline phase.

A glass ceramic is generally defined as a material obtained by crystallizing glasses. Sea-island and core-shell structures including crystalline nanoparticles have been proposed as possible models of glass ceramics. However, the size and morphology of crystals in an amorphous matrix have not been clarified yet. Although various experiments have been conducted so far to reveal the structure of Li_2_S–P_2_S_5_ solid electrolytes^13–16^, there have been no direct observations of the microstructure of the corresponding glasses and glass ceramics. In this study, we carried out direct observation and structural analysis for the 75Li_2_S·25P_2_S_5_ and 80Li_2_S·20P_2_S_5_ solid electrolytes using transmission electron microscopy (TEM). Notably, Li_2_S–P_2_S_5_ glasses composed of approximately compositions 75 mol% Li_2_S exhibited great chemical stability. H. Muramatsu. *et al*. showed that in the Li_2_S–P_2_S_5_ system, 75Li_2_S·25P_2_S_5_ glasses generated the least amount of H_2_S when exposed to air^17^. This excellent chemical stability provide the best experimental condition for *in-situ* TEM observation of Li_2_S–P_2_S_5_ solid electrolytes because the sulfide-based solid electrolyte is highly sensitive to electron beam irradiation.

## Results and Discussion

**Figure 1:**
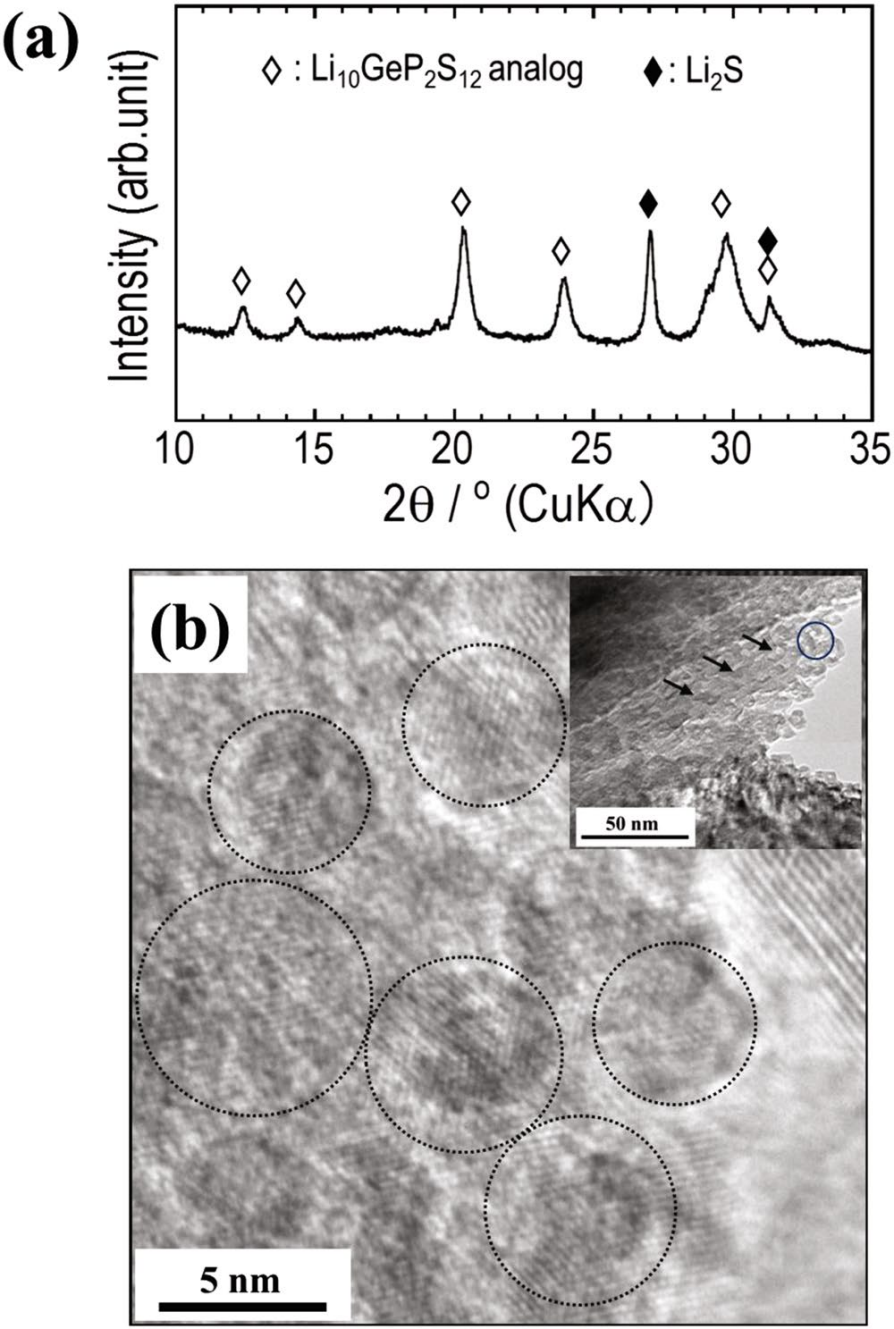
(**a**) The XRD pattern of the 80Li_2_S·20P_2_S_5_ glass ceramics obtained by heat treatment. (**b**) HR-TEM image indicating the microstructure of the 80Li_2_S·20P_2_S_5_ glass ceramics, together with a BF image in the inset. The image was obtained from the area indicated by the blue circle in the BF image.

We first examined the microstructure of the 80Li_2_S·20P_2_S_5_ glass ceramics obtained by heat-treatment at 240 °C. Fig. 1(a) and (b) shows the XRD pattern and high resolution (HR) TEM image of the 80Li_2_S·20P_2_S_5_ glass ceramics. The inset of Fig. 1(b) shows a bright field (BF) image indicating the morphology of 80Li_2_S·20P_2_S_5_ glass ceramics. Characteristic speckled contrasts could be observed, as indicated by small arrows in the BF image. These contrasts are attributed to the aggregation of crystalline nanoparticles. The HR-TEM image (b) was obtained from the area indicated by the circle in the BF image. In the XRD pattern (a), the profiles due to a Li_10_GeP_2_S_12_ (LGPS) analog crystal (Li_3.25_P_0.95_S_4_) phase were detected. In the HR-TEM image (b), lattice fringes were observed in the sample. Notably, crystalline nanoparticles with an average size of about 5 nm were present in an amorphous matrix and connected to each other, as indicated by dotted circles. These nanoparticles appear to be LGPS analog crystals. This indicates that precipitation and connection of LGPS analog crystalline nanoparticles in an amorphous matrix contributes to a high ionic conductivity of more than 10^−3^ S cm^−1^ in the 80Li_2_S·20P_2_S_5_ glass-ceramics^10^.

**Figure 2:**
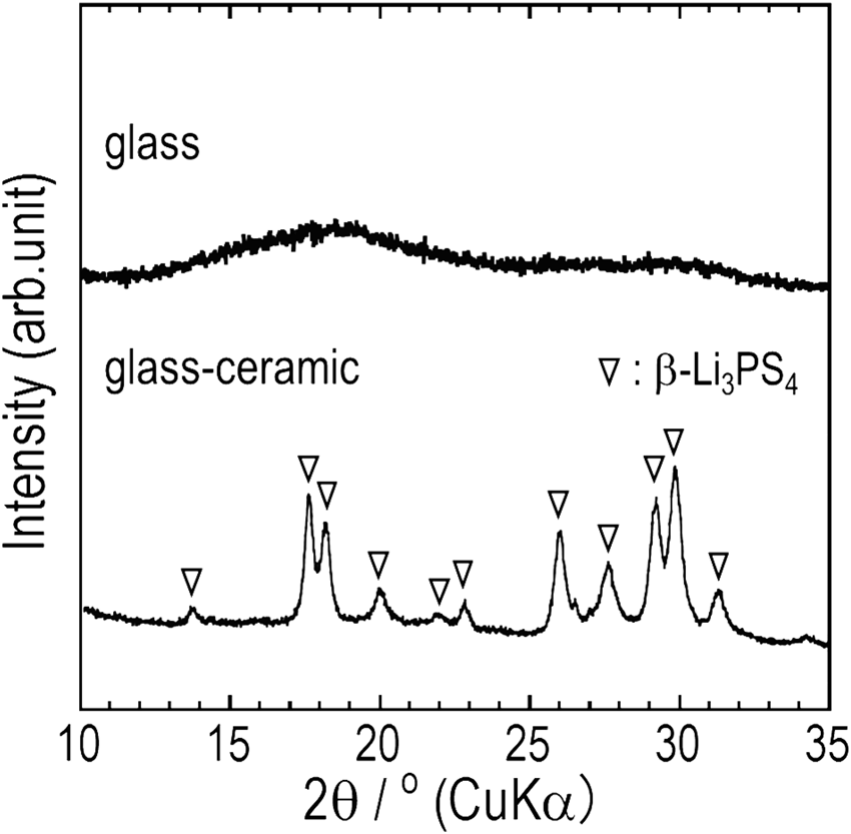
The XRD patterns of the 75Li_2_S·25P_2_S_5_ glass and glass ceramic.

**Figure 3:**
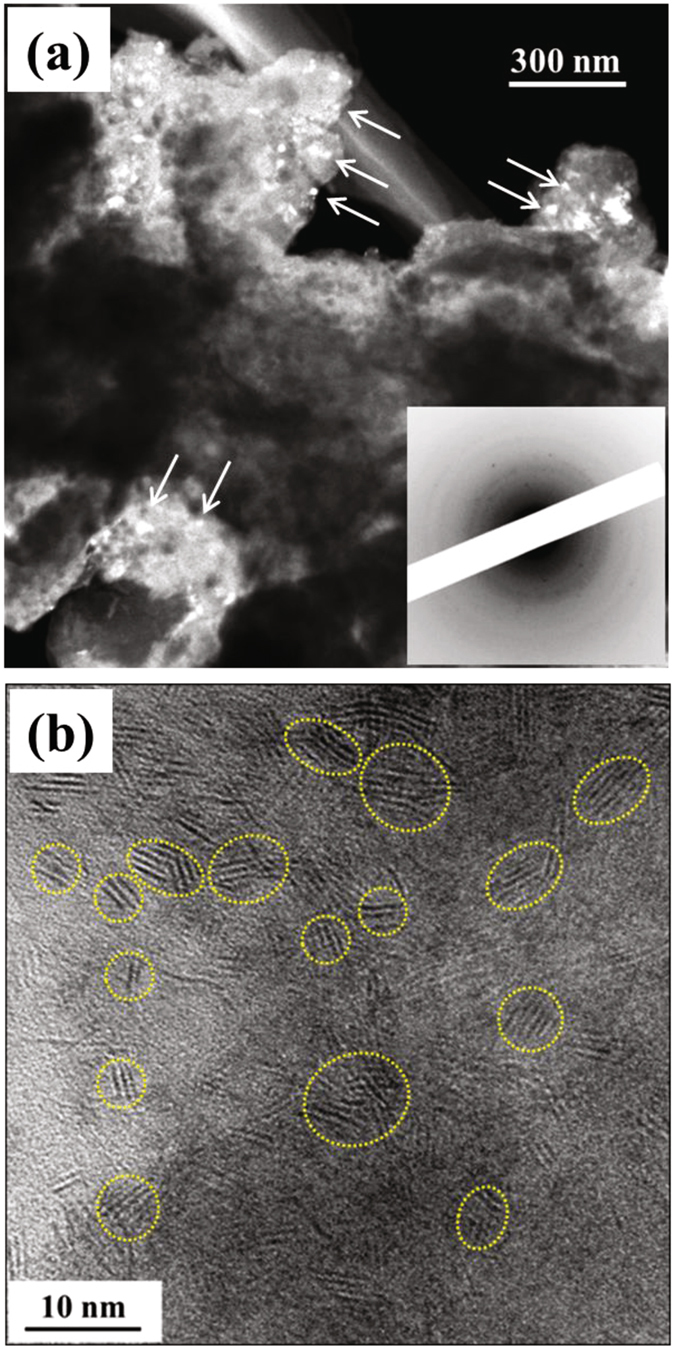
The DF image (**a**) and HR-TEM image (**b**) indicating the microstructure of the 75Li_2_S·25P_2_S_5_ glasses, together with the corresponding ED pattern in the inset.

Next, the microstructure of the 75Li_2_S·25P_2_S_5_ glasses and glass ceramics were investigated. First, we confirmed the crystal structures of the 75Li_2_S·25P_2_S_5_ glasses and glass-ceramics by powder XRD experiments. The glass-ceramic sample was prepared by heating precursor 75Li_2_S·25P_2_S_5_ glasses at 210 °C. Figure 2 shows the XRD patterns of the 75Li_2_S·25P_2_S_5_ glasses and glass-ceramics at room temperature. The as-prepared glass sample after mechanical milling was intrinsically amorphous. On the other hand, the glass-ceramic sample exhibited XRD patterns that are attributable to the $\beta$-Li_3_PS_4_ crystal^6^. To further understand the microstructure and crystallization behavior in the 75Li_2_S·25P_2_S_5_ glasses, *in-situ* TEM observation were carried out. Figure 3(a) and (b) show DF and HR images of the microstructure of the 75Li_2_S·25P_2_S_5_ glasses. The corresponding electron diffraction (ED) pattern is shown in the inset of Fig. 3(a). Typical halo rings were observed in the ED pattern. This indicates that the spatially-averaged structure of 75Li_2_S·25P_2_S_5_ glasses are characterized by an amorphous state. In the DF image (a), however, crystallized regions exhibiting bright contrasts were visualized, as indicated by arrows. The average size of crystalline nanoparticles was approximately 5 to 20 nm. In addition, crystalline nanoparticles were likely to scatter over the sample without connecting. This image is entirely different from the 75Li_2_S·25P_2_S_5_ glass ceramics, which will be discussed later. In the HR-TEM image (b), furthermore, crystalline nanoparticles with an average size of approximately 5 nm are randomly distributed in an amorphous matrix, as indicated by yellow dotted circles. That is, in spite of a spatially-averaged amorphous structure, crystalline nanoparticles were present in the 75Li_2_S·25P_2_S_5_ glasses.

**Figure 4:**
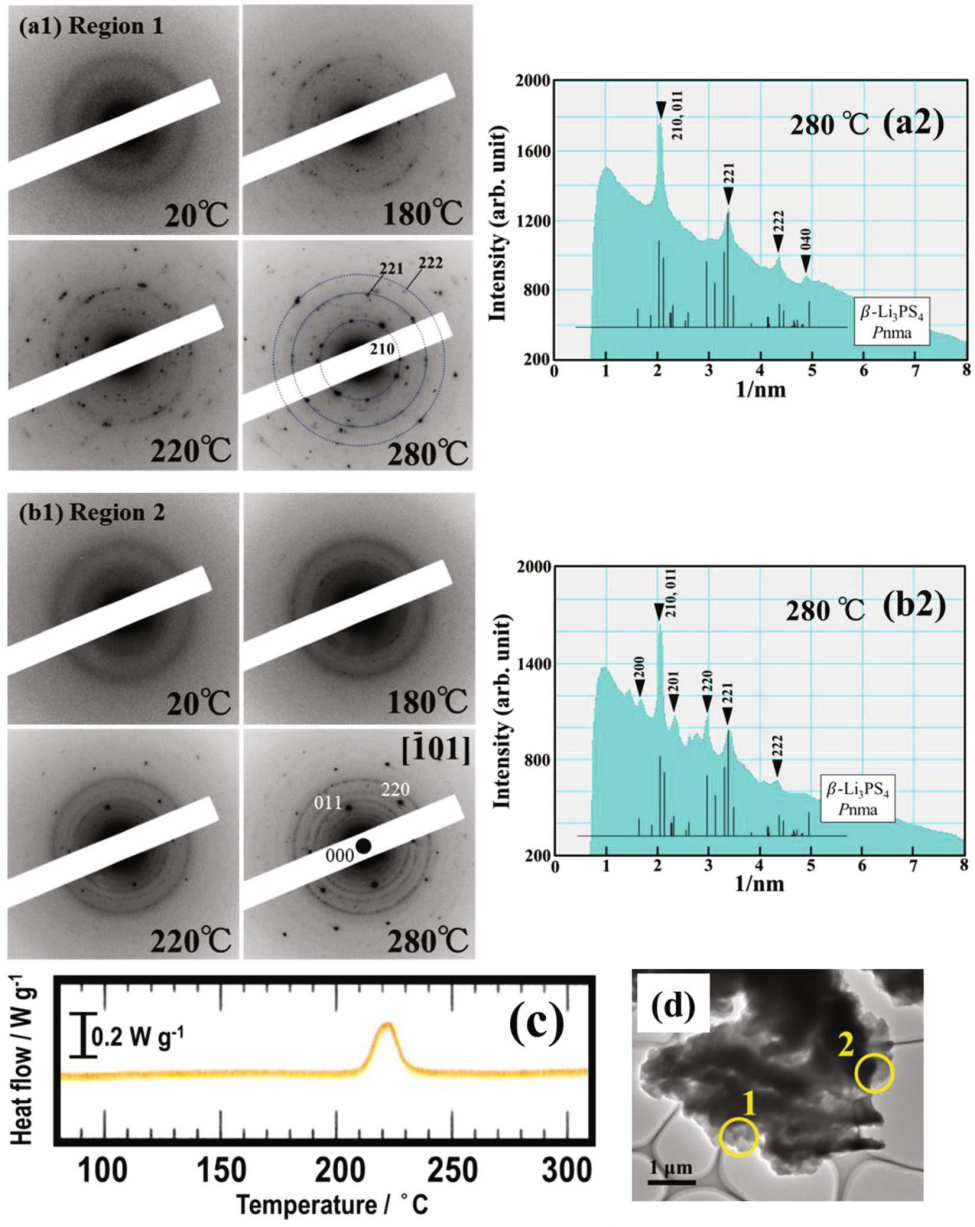
Series of ED patterns (**a1**) and (**b1**) as a function of temperature in the milled 75Li_2_S·25P_2_S_5_ glasses, which were obtained from regions 1 and 2, respectively, as indicated by yellow circles in the BF image (**d**). The integrated intensity profiles (**a2**) and (**b2**) were constructed from the ED pattern at 280 °C in regions 1 and 2, respectively. The DSC curve of the 75Li_2_S·25P_2_S_5_ glasses is shown in (**c**).

To understand the crystallization behavior of the 75Li_2_S·25P_2_S_5_ glasses, we conducted *in-situ* TEM observations of the heating process. Figure 4 shows variations of ED patterns as a function of temperature, together with the corresponding BF image, DSC curve and integral intensity profiles of the ED patterns at 280 °C. A series of ED patterns in Fig. 4(a1) and (b1) were respectively obtained from regions 1 and 2, as indicated by yellow circles in the BF image (d). The integral intensity profiles of the ED pattern at 280 °C in regions 1 and 2 are shown in Fig. 4 (a2) and (b2), respectively. In the initial state at 20 °C, the ED patterns in regions 1 and 2 exhibited halo rings, which indicate an amorphous state. At region 1, crystallization was detected at approximately 180 °C and, remarkably, proceeded above 200 °C with increasing temperature, as seen in Fig. 4 (a1). To identify the precipitated crystalline phase, we analyzed the integral intensity profiles of the ED pattern at 280 °C. As seen in Fig. 4 (a2), the peak positions of the intensity profile almost correspond to the XRD profiles for orthorhombic $\beta$-Li_3_PS_4_ with the space group *P*nma^6^. When we investigated the crystallization behavior of many other particles, we found that a polycrystalline state similar to region 1 frequently appeared. At region 2, on the other hand, crystallization could be observed at approximately 220 °C, as seen in Fig. 4 (b1). The intensity profiles shown in Fig. 4 (b2) approximately correspond to the XRD profiles of the $\beta$-Li_3_PS_4_ crystal. The ED pattern at 280 °C indicates the precipitation of a single crystalline particle having the same crystal orientation. This crystallization behavior was relatively minor. Based on the analysis of the ED pattern at 280 °C, the crystal orientation was parallel to the $[\bar{1}10]$ direction in the orthorhombic *P*nma structure. Thus, the crystallization behavior of the 75Li_2_S·25P_2_S_5_ glasses can be characterized by the precipitation of a $\beta$-Li_3_PS_4_ crystalline phase. This is compatible with the XRD pattern in Fig. 2. These crystallization behaviors are compared with the DSC curve in Fig. 4(c). The exothermal peak observed at around 220 °C is attributable to the transition from glass to the $\beta$-Li_3_PS_4_ crystal.

**Figure 5:**
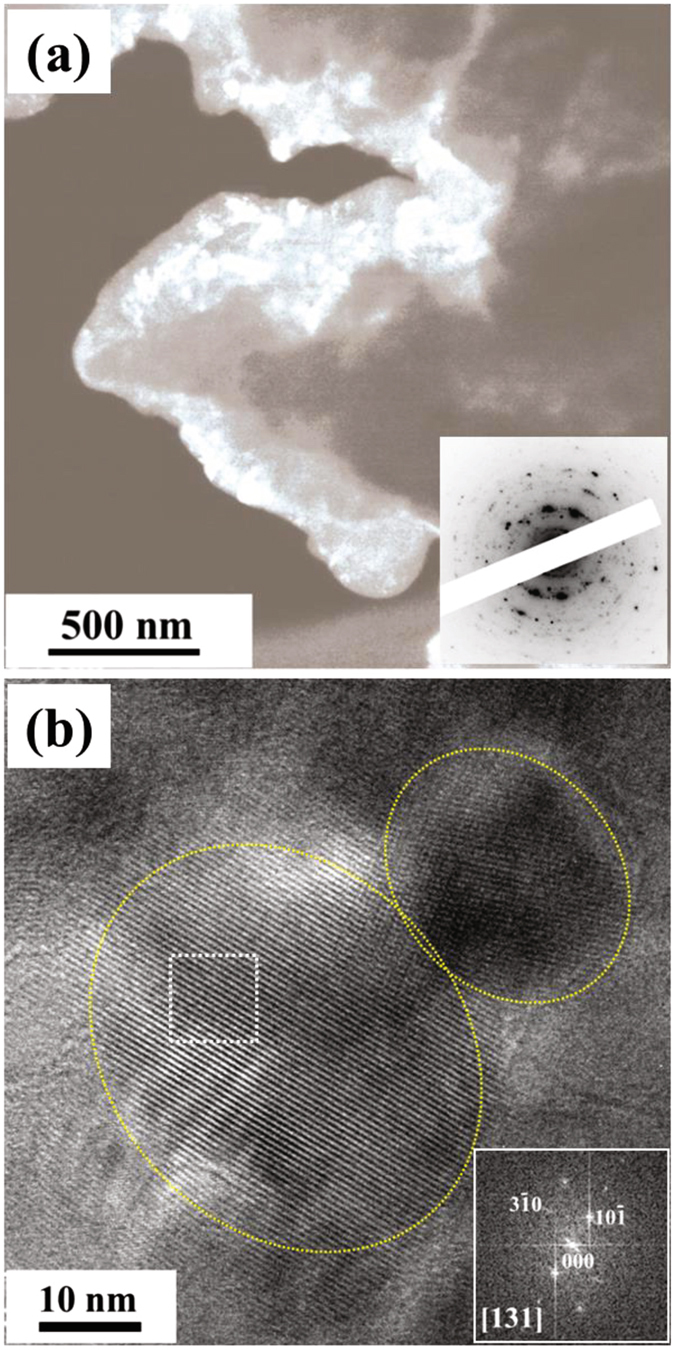
The DF image (**a**) and HR-TEM image (**b**) indicating the microstructure of the the 75Li_2_S·25P_2_S_5_ glass ceramics, together with the corresponding ED and FFT pattern in the inset of (**a**) and (**b**), respectively. The FFT pattern was obtained from the dotted square regions in (**b**).

To reveal the microstructure of the 75Li_2_S·25P_2_S_5_ glass ceramics, we carried out *in-situ* TEM observation at 210 °C. Figure 5(a) and (b) show DF and HR-TEM images, together with the corresponding ED pattern in the inset of Fig. 5(a). In the DF image (a), numerous crystallized regions can be observed as bright contrasts compared to the glasses. On average, the sizes of crystalline nanoparticles were approximately 30 to 60 nm. Crystalline nanoparticles were likely to connect to each other. In the ED pattern, Debye-Scherrer rings consisting of spots were observed, together with weak halo rings. Based on the analysis of the integral intensity profile, the crystal structure was confirmed to be $\beta$-Li_3_PS_4_. In the HR-TEM image (b), lattice fringes can be clearly observed and crystalline particles with an average size of approximately 30 nm are adjacent to each other, as indicated by yellow dotted circles. To understand the crystal structure of the crystalline particles, we carried out Fast Fourier transform (FFT) calculations for the nanoscale local regions with an area of approximately 10 × 10 nm^2^. The FFT pattern obtained from the region indicated by a dotted white square is shown in the inset of Fig. 5(b). In the FFT pattern, the ED pattern with the [131] incidence from the orthorhombic *P*nma structure was reproduced. Based on these patterns, the 75Li_2_S·25P_2_S_5_ glass ceramics were characterized by the aggregation of $\beta$-Li_3_PS_4_ crystalline particles in an amorphous matrix.

In the present study, we examined microstructures of Li_2_S–P_2_S_5_ glasses and glass ceramics via *in-situ* TEM observation. HR-TEM and DF images revealed that 75Li_2_S·25P_2_S_5_ glasses were characterized by an amorphous state, in which nano-sized crystals were randomly distributed. These nano-sized crystals are shown in Fig. 3(b) and may play an essential role as a nucleus for crystallization. On the other hand, 75Li_2_S·25P_2_S_5_ and 80Li_2_S·20P_2_S_5_ glass ceramics were characterized by an aggregation of crystalline nanoparticles. Notably,$\beta$-Li_3_PS_4_ and LGPS analog crystalline nanoparticles, which are responsible for high ionic conductivity, connected to each other in an amorphous matrix. This implies that ionic conduction in Li_2_S–P_2_S_5_ glass ceramics is due to the connection between precipitated crystalline nanoparticles. In addition, *in-situ* TEM observation during heating revealed that the crystallization started at around 200 °C and the $\beta$-Li_3_PS_4_ crystalline phase precipitated. This suggests that the exothermal reaction in the 75Li_2_S·25P_2_S_5_ glasses is due to crystallization.

## Conclusion

We examined the microstructures of Li_2_S–P_2_S_5_ glasses and glass ceramics via TEM. *In-situ* TEM observation at room temperature revealed that the glasses could be identified as an amorphous state studded with crystalline nanoparticles; whereas, the glass ceramics were characterized by the aggregation of $\beta$-Li_3_PS_4_ and LGPS analog crystalline particles exhibiting high ionic conductivity. To reveal the crystallization behavior of the glasses, we investigated the crystallization process in 75Li_2_S·25P_2_S_5_ glasses. We found that $\beta$-Li_3_PS_4_ crystals started to precipitate at approximately 200 °C in an exothermal reaction. Based on these data, the precipitation and connection of $\beta$-Li_3_PS_4_ and LGPS analog crystalline nanoparticles contribute to high ionic conductivity.

## Method

### Sample preparation

The Li_2_S–P_2_S_5_ glass solid electrolytes were prepared by mechanical milling technique. Li_2_S (Idemitsu Kosan Co., >99.9%) and P_2_S_5_ (Aldrich, 99%) initial crystalline powders was used to prepare the 75Li_2_S·25P_2_S_5_ and 80Li_2_S·20P_2_S_5_ (mol %) glass sample. The mechanical milling process was conducted at room temperature by a planetary ball mill apparatus (Fritsch Pulverisette 7) consisting of an Al_2_O_3_ pot (volume 45 ml) and Al_2_O_3_ balls (10 mm in diameter)^11^. The milling time was 20 h and the rotation speed was 370 rpm. All the processes were conducted in a glove box filled with dry argon gas (less than 1 ppm water). The 80Li_2_S·20P_2_S_5_ glass-ceramic sample was obtained by crystallizing the mechanically milled 80Li_2_S·20P_2_S_5_ glass via heat-treatment at 240 °C for 2 h. To identify the precipitated crystal phase, X-ray diffraction (XRD) measurements were performed using a diffractometer (Rigaku Ultima IV) with CuKα radiation. Thermal behavior of the glass solid electrolytes was examined with a differential scanning calorimeter (DSC, Rigaku Co., model DSC 8231). The Li_2_S–P_2_S_5_ glass sample for the DSC measurements was put into the stainless-steel pan, which was then crimp-sealed. The DSC measurements were carried out at the heating rate of 5 °C min^−1^ from room temperature to 400 °C.

### TEM observation

*In-situ* TEM observations were carried out using JEM-2100F field-emission-type TEM systems with acceleration voltages of 200 kV in the temperature range from 20 to 300 °C. Samples were mounted on an amorphous carbon film supported by a Cu grid for TEM observation, which was then attached to a TEM holder in a dry Ar-filled glove box. During observation, we used a double-tilt vacuum transfer TEM holder (Gatan model 648) to prevent the sample from being exposed to air. The vacuum degree in TEM was approximately 1.0 × 10^−5^ Pa. Since the Li_2_S–P_2_S_5_ solid electrolytes are very sensitive to the electron beam irradiation, the electron beam was not converged on the Li_2_S–P_2_S_5_ sample as much as possible to avoid destroying it. In addition, to realize the optimum intensity of the electron beam for observations of the Li_2_S–P_2_S_5_ solid electrolytes, the size of the condenser aperture was set to be 40 μm in diameter. Features of the microstructure and crystallization behavior of Li_2_S–P_2_S_5_ solid electrolytes were examined by taking high resolution (HR) TEM images, dark field (DF) images, and corresponding electron diffraction (ED) patterns with a 14-bits CCD camera. To prevent damage to the sample by the electron beam, the exposure time was within 2 seconds. For identification of the precipitated crystalline phase from ED patterns, we utilized a computer program called “Process Diffraction”. In Process Diffraction, digital ED patterns can be transformed into integral intensities as a function of scattering angle, producing the intensity profile such as a usual XRD pattern^18–21^. By comparing the obtained ED patterns with XRD profiles, it was possible to identify the precipitated crystalline phase.
